# Health and Employment after Fifty (HEAF): a new prospective cohort study

**DOI:** 10.1186/s12889-015-2396-8

**Published:** 2015-10-19

**Authors:** Keith T. Palmer, Karen Walker-Bone, E. Clare Harris, Cathy Linaker, Stefania D’Angelo, Avan Aihie Sayer, Catharine R. Gale, Maria Evandrou, Tjeerd van Staa, Cyrus Cooper, David Coggon

**Affiliations:** MRC Lifecourse Epidemiology Unit, University of Southampton, Southampton, UK; ARUK-MRC Centre for Musculoskeletal Health and Work, University of Southampton, Southampton, UK; Centre for Research on Ageing, University of Southampton, Southampton, UK; Academic Geriatric Medicine, Faculty of Medicine, University of Southampton, Southampton, UK; NIHR Southampton Biomedical Research Centre, University of Southampton and University Hospital Southampton NHS Foundation Trust, Southampton, UK; NIHR Collaboration for Leadership in Applied Health Research and Care: Wessex, Southampton, UK; Newcastle University Institute for Ageing and Institute of Health & Society, Newcastle University, Newcastle upon Tyne, UK; Farr Institute, University of Manchester, Manchester, UK

**Keywords:** Ageing population, Older worker, Retirement, CPRD

## Abstract

**Background:**

Demographic trends in developed countries have prompted governmental policies aimed at extending working lives. However, working beyond the traditional retirement age may not be feasible for those with major health problems of ageing, and depending on occupational and personal circumstances, might be either good or bad for health. To address these uncertainties, we have initiated a new longitudinal study.

**Methods/design:**

We recruited some 8000 adults aged 50–64 years from 24 British general practices contributing to the Clinical Practice Research Datalink (CPRD). Participants have completed questionnaires about their work and home circumstances at baseline, and will do so regularly over follow-up, initially for a 5-year period. With their permission, we will access their primary care health records via the CPRD. The inter-relation of changes in employment (with reasons) and changes in health (e.g., major new illnesses, new treatments, mortality) will be examined.

**Discussion:**

CPRD linkage allows cost-effective frequent capture of detailed objective health data with which to examine the impact of health on work at older ages and of work on health. Findings will inform government policy and also the design of work for older people and the measures needed to support employment in later life, especially for those with health limitations.

**Electronic supplementary material:**

The online version of this article (doi:10.1186/s12889-015-2396-8) contains supplementary material, which is available to authorized users.

## Background

During recent decades, the proportion of people in Western countries aged 50 years or older has steadily grown, and by 2050, it is expected that about 30 % of the European population will be aged >65 years. This demographic trend generates an economic imperative for people to remain in work to older ages, especially in countries where reproduction and immigration rates are low. In response, governments have developed policies to boost labour force participation among older workers [1]. The UK government, for example, has raised the State pension age, abolished the default retirement age, legislated to remove age and disability discrimination in the workplace, and implemented other policies [2, 3] to maximise employment. At the same time, increasing numbers of people are intent on working longer to build savings for retirement in the face of personal indebtedness, higher costs and taxes, and diminishing returns on savings and pensions. A steady rise in the proportion of men and women working beyond the traditional retirement age has ensued [4] and this trend is likely to continue [5].

Work at older ages may confer psychological benefits (for example, sustained motivation, sense of purpose and achievement, social engagement, and mental stimulation), and physical benefits (through maintained mobility and muscle strength) [6], while involuntary job loss may precipitate psychological ill-health. Additionally, work may provide the wherewithal to support self and dependants and improve social cohesion in communities [7]. Set against this, older workers may struggle with the physical and psychological demands of work [6], and in principle their greater prevalence of illness and use of medication could pose higher risks of occupational injury [8, 9]. Moreover, planned retirement may carry tangible health benefits of its own, especially when desired and expected [10, 11], and foregoing it may sometimes be bad for psychological health. An influential report for the Department for Work and Pensions in the UK has concluded that work is ‘generally good’ for health [12]. However, few data were available on the impact of deferred retirement in older workers, or on potential effect modifiers such as type of job surrendered (e.g., casual vs. permanent, physically or mentally demanding vs. less so, rewarding vs. disliked) [13], or the circumstances of job loss (e.g., involuntary redundancy vs. normal retirement with adequate financial security) [12]. There is thus uncertainty about the overall health implications of policies to extend working life and maximise employment at older ages. It is quite likely outcomes will vary according to circumstances, and limited data support the notion of effect modification by age and other factors [11, 12, 14, 15]. Presently, however, it remains unclear whether continuing work to older ages produces net benefits or harm to health and in what circumstances. Knowing the factors that predict a favourable outcome will become increasingly important in designing suitable work and social support for older workers.

A second major area of uncertainty concerns the extent to which common health problems in older people limit their participation. For example, among disorders affecting the musculoskeletal system, some become more common and severe at older ages (e.g., osteoarthritis) and others may become more limiting (e.g., soft tissue rheumatism, disorders of the back, neck, upper limbs and knee cartilage), with the potential to reduce late-career capacity for work [16]. The impact may especially be felt by workers with other concurrent medical problems that might otherwise be compatible with working [17]. Better understanding of the impact of disease and illness on employment at older ages, and the factors that make it easier (or more difficult) for those with health problems to remain in safe productive work, is important for public health policy, needed to aid the design of jobs that better accommodate older workers with health limitations. Again, the context is likely to be important, some work circumstances being more forgiving of health limitations than others, and some health limitations being more amenable to accommodation in the workplace. Understanding is required of how much work outcomes vary by diagnosis and environment, and which types of intervention are needed and for whom.

A third uncertainty, given the rising prevalence of age-related disorders and their treatments in modern workforces, is the associated risk to physical safety and the jobs that older workers can safely perform. A systematic review of health and risk of occupational injury [8] highlighted the paucity of data and the difficulty managers will have in setting evidence-based employment policies.

A fourth area bearing investigation concerns the impact that social and financial factors have on retirement intentions (e.g., affordability, other commitments and interests), and how this varies by health status and circumstances of employment.

Finally, effective planning to maximise work opportunities at older ages requires information on the descriptive epidemiology of ageing and adverse employment outcomes. For example, it would be helpful to know: how often middle-aged workers struggle to cope at work; how often they quit a job for medical reasons and which disorders are most often responsible; the levels of sickness absence in older workers from the general population and its leading causes; how well medical factors and indices of mental and physical health predict sickness absence and job loss; the likelihood that an older adult who quits a job for medical reasons will find re-employment, and how this varies by reason for job loss; how patterns of job loss vary by type of work and how much they are modified by workplace psychosocial and physical conditions and access to rehabilitation services; and how the demands and perceived rewards of work, and employers’ support, bear on retirement intentions and work retention. Only limited data are currently available to answer these questions, but all require answers urgently, given the changing demographics in modern workforces.

As a precursor to the development of guidance for employers and its assessment through intervention studies, we have been funded by Arthritis Research UK, the Medical Research Council, and the Economic and Social Research Council (ESRC) to establish a new cohort investigation of ageing and employment transition called the **H**ealth and **E**mployment **A**fter **F**ifty (HEAF) study. In this report we describe the aims of the HEAF study, its methods of recruitment and the participation rates at baseline, the information being collected and data sources, and our plans to date for follow-up, analysis and related field work.

## Objectives

The aims of the HEAF study are:

1. To assess the health benefits and risks of remaining in work at older ages and their predictors (health as an outcome), and thereby the potential health impact of policies to extend working life and maximise employment in later working life; to identify occupational, social and personal co-factors which modify this relationship, as possible targets for intervention.
2. To assess the impact of health on employment outcome and lost work time (health as an exposure) - e.g., the impact of musculoskeletal illness at older ages on work capability, employment status, and job retention, to enable the development of interventions that support extended working life.

The study will also lend itself to:

3. Assessing the effect of common health problems of ageing on risk of workplace injuries (health as an exposure with injury as an outcome), and therefore refined risk assessment in the job placement of older workers.
4. Mapping the descriptive epidemiology of ageing and employment transitions, including factors that may promote or hinder extended working.

## Methods

### Ethical approval

The protocol “Health risks and benefits of extended working life” (RGO 8569) was approved by the National Research Ethics Service Committee North West-Liverpool East (REC reference 12/NW/0500) and by the Independent Scientific Advisory Committee of the Clinical Practice Research Datalink (reference 12_054R2), as well as being adopted by the Hampshire and Isle of Wight NIHR Clinical Research Network (reference 103258).

### Study design

The HEAF investigation is an observational prospective cohort study.

### The CPRD database

To facilitate the collection of health-related data, the study sample has been recruited from patients registered with general practices contributing data to the Clinical Practice Research Datalink (CPRD). The CRPD, formerly known as the GPRD, was originally established in 1987 to enable post-marketing surveillance of drug safety, and has since been maintained as a research resource by the Medicines and Healthcare Products Regulatory Agency (MHRA), an executive agency of the English Department of Health. The CPRD provides a log of all medical consultations in primary care and hospital associated with significant events, illnesses, or medical activity (diagnosis, referral, prescription, etc.) among patients from participating general practices. Data are obtained on some five million patients from about 590 participating general practices throughout the UK (about 6 % of the national population, almost all of whom are registered with general practices) [18], uploaded regularly in anonymised form, and checked for completeness (>97 %) and validity (deemed high in several external audits of selected end-points [19–21]). Events are linked at the individual level via a unique identifying code number. Although health information is well captured, other variables (such as employment and job transitions, occupational demands and support, attitudes to work and retirement, personal, social and demographic characteristics, health behaviours and beliefs, self-perceived health and retirement expectations) are not. These are therefore ascertained in the HEAF study by means of a postal questionnaire.

### Recruitment

#### Practices

In 2012, the CPRD advertised the HEAF study to all practices in England contributing data to its database. (The CPRD collects data also from Scotland, Wales and Northern Ireland, but geographical restriction was employed to allow later linkage with English databases that record hospital inpatient and outpatient care (Hospital Episode Statistics), as well as mortality and cancer incidence (Health and Social Care Information Centre)). Practices that volunteered to assist recruitment into the HEAF study were made known to the research team and all that did so became foci of recruitment, until the target sample size was met.

**Fig. 1:**
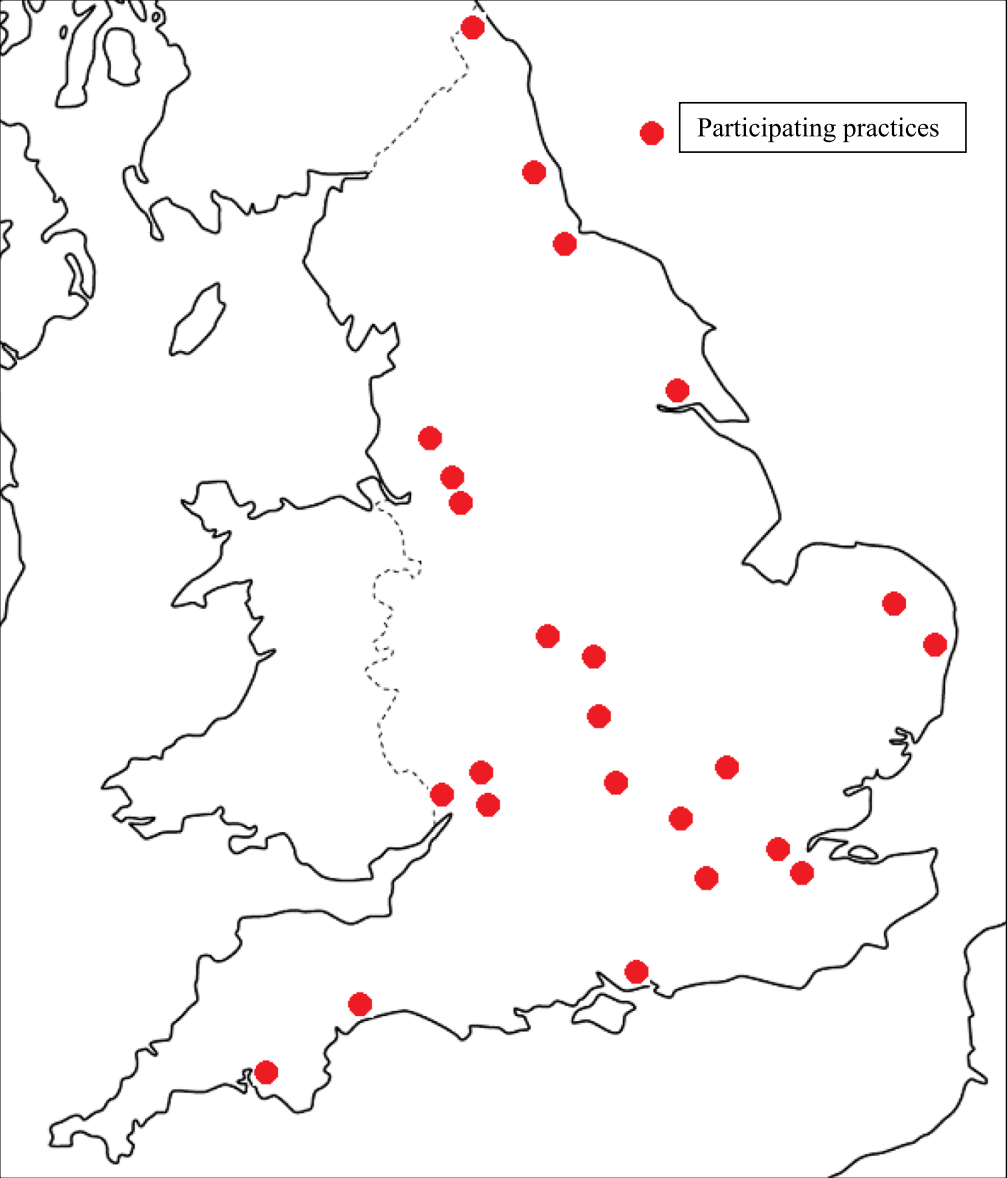
Location of practices participating in the HEAF study

In all, 24 general practices finally contributed to the sampling frame (during Jan 2013 to June 2014). These offered a good geographical spread, with recruitment from the South, Midlands and North of England (Fig. 1). (There was no requirement that the distribution of respondents’ occupations should be nationally representative, but geographical dispersion was deemed desirable as unemployment rates and patterns of illness behaviour and consulting are liable to vary between regions.)

#### Participants and recruitment

All patients born between 1948 and 1962 (target age band 50–64 years) who were registered with the participating practices were eligible to be recruited, although general practitioners (GPs) were asked to review the sampling lists before mailing and to exclude patients whom they thought should not be approached (e.g., because of terminal illness or recent bereavement). Mailings were conducted initially by the practices (between January 2013 and June 2014). A single invitation was issued without reminder. To safeguard the privacy of non-participants, contact details were withheld from the researchers until those who agreed to participate returned their baseline questionnaire, their written consent (relating to follow-up and accessing their medical records) and their contact information (Table 1). Methods of recruitment were piloted and response rates were assessed in two of the practices before recruitment was rolled out to the remainder.

**Table 1:** The baseline recruitment protocol

1) The CPRD advertised the study to practices already participating in CPRD data collection; volunteer practices were identified and made known to the research team. Their practice managers were approached.
2) The research team provided each practice with a model letter from GP to patient introducing the study and the researchers. One generic letter was signed by a doctor in the practice and returned to the research team for copying and inclusion in mailings to patients.
3) The CPRD sent each practice manager an electronic file listing the CPRD-coded identifier and a special study code number for each patient from the practice eligible to take part in the study (those born between 1948 and 1962 inclusive).
4) A member of the practice staff added the name and address of each patient to this file.
5) The GP excluded any patients from the mailing list whom he or she felt should not be approached (e.g., because of terminal illness or recent bereavement).
6) A member of the practice staff printed an address label for each of the remaining patients, including the study code number and the name and address.
7) The research team delivered to each practice a set of sealed envelopes with postage pre-paid, each marked with a study code number.
8) Practice staff attached the appropriate address label to each envelope and mailed them. Envelopes for patients withdrawn from the study were counted and destroyed.
9) Questionnaires were returned directly to the research team. Consent to baseline self-completed information was signified by return of a questionnaire. Additionally, participants were asked to complete and return a signed consent to the further stages of data linkage and postal follow-up, and to provide their name and contact details to the research team to enable follow-up without need for further involvement of the practices.

### Baseline questionnaire

The baseline questionnaire (Additional file 1) was tested for ease of completion in 10 clerical staff of comparable age to the target study population. All items on the questionnaire were completed by all respondents; completion times ranged from 10 to 25 min with a median of 17 min, eight of the individuals taking less than 20 min in total.

The questionnaire covered the following main domains: demographic and anthropometric characteristics; current work status; content and characteristics of paid work; physical and psychosocial demands of work; feelings about work, financial status and retirement expectations and plans; leisure and social activities; and selected items on health. The principal variables in each domain are listed in Table 2. Below we comment on the properties of the key measures, several of which are widely used standards, and our intended analytic treatment of them.

**Table 2:** Main domains and variables on which information was collected at baseline

Domain	Variables
Demographic and anthropometric characteristics	Age, sex, height and weight, marital status, ethnic origin, qualifications and education, household composition
Current work status	Employed, self-employed, unemployed, or retired; more than one job; left last job for a health reason; receiving an ill-health pension
Content and characteristics of paid work	Main occupation, length of service, pattern of work (e.g., salaried vs. piece work, permanent vs. temporary, shift and night working, income protection in illness, flexibility of working hours) and employer’s size; physical demands of work (e.g., regular kneeling, climbing, digging, lifting, and standing)
Perceptions about work	Psychosocial demands, support from colleagues or manager, decision latitude, self-assessed ability to cope with the physical and mental demands of work; worry or anger about work; other feelings about work, e.g., satisfaction with work schedule, pay, and the job overall, conflicts at work and relational justice, perceived job security
Perceptions about retirement	Retirement expectations, ambitions and plans (e.g., expected and ideal retirement age)
Financial status	Housing tenure, affordability of consumer durables, contribution to total household income, pension provision, financial responsibility for others
Social	Leisure and social activities; smoking and alcohol history; workplace friendships; caring and voluntary responsibilities
Health	Self-rated health (SRH) [22]; an abridged Sleep Problems Scale [23]; five items from the somatising subscale of the Brief Symptom Inventory (BSI) [24]; the Center for Epidemiologic Studies Depression Scale (CES-D) [25], the Warwick-Edinburgh Mental Well-being Scale (WEMWBS) [26]; certain items on frailty (based on the Fried frailty index [27]), and hearing and memory impairments; chronic regional pain in the past 12 months; sickness absence in the past 12 months; presenteeism

#### Occupational outcomes

Questions were posed about: current employment status (with current occupation coded according to the Standard Occupational Classification 2010 (SOC 2010) [22], allowing a determination of social class); and, among those who were retired or unemployed, about quitting an earlier job for a health reason or receiving an ill-health pension.

Among those in work, information was collected on sickness absence in the past 12 months (overall and related to musculoskeletal pain); on having to cut down on work activities because of ill-health; and on perceived coping with workplace demands, as well as expectations of future coping.

#### Measures of health

Self-rated health (SRH), which is known to predict mortality and morbidity [23], was assessed using the question: “In general would you say your health is…excellent/very good/good/fair/poor”; for most purposes we plan to combine the response categories ‘good and ‘very good’, and also those for ‘fair’ and ‘poor’ to create a scale with three levels.

Somatising tendency was measured using questions from the Brief Symptom Inventory (BSI) [24] which asked about distress from five common physical symptoms (nausea, faintness or dizziness, chest pain, hot or cold spells and breathing difficulties) during the past 7 days. Subjects were classified according to the number of such symptoms reported as causing at least moderate distress, a measure which has been shown to predict incident and persistent regional pain [25, 26].

Depression was assessed through the 20-item Center for Epidemiologic Studies Depression Scale (CES-D), which measures frequency of symptoms of depression over the past 7 days on a four-point ordinal scale (<1 day = 0 through to 5–7 days = 3) [27] and covers nine different components, including depressive mood, feelings of guilt and worthlessness, psychomotor retardation, loss of appetite, and sleep disturbance; points are summed (with scores inverted for four of the items), a cut-off score of 16 (in a range of 0 to 60) often being taken as indicative of “significant” or “mild” depression. The scale is widely used and has high internal consistency and adequate test-retest repeatability and concurrent and discriminant validity.

We also included the 14-question Warwick-Edinburgh Mental Well-being Scale (WEMWBS), which assesses the frequency of feelings and thoughts about positive well-being over the previous two weeks on a five-point ordinal scale (‘none of the time’ = 1 through to ‘all of the time’ = 5); points are summed to give a scale range of 14 to 70, population scores being normally distributed with a mean of about 50 points. The WEMWBS has been shown to have acceptable internal consistency, test-retest repeatability, and content and construct validity [28, 29].

The 28-item Sleep Problems Scale of Jenkins et al. has established test-retest reliability and internal consistency [30]. We selected four principal questions from it concerning difficulty in falling asleep, staying asleep, waking too early, and feeling unrefreshed; these can be scored on a four-point scale, ranging from ‘no problem’ to ‘severe problem’, reference data being available from a large British population-based study of incident and persistent insomnia [31].

The Work Ability Index (WAI) [32] comprises self-reported items on current work ability (relative to a lifetime best and the physical and mental demands of work), expectations of work capacity in two years’ time, presenteeism, sickness absence in the past year, psychological resources (enjoyment of daily tasks, optimism about the future) and tally of diagnosed diseases. The WAI has adequate test-retest reliability [33] and is predictive of future work incapacity and disability pensioning [34]. Questions 44–46, 89, 91, 39, and 84 in Additional file 1 (when linked with CPRD data on physician’s diagnoses) will provide proxy information on most aspects of work ability, similar to the WAI, although there is no intention to calculate a WAI score per se.

The Fried frailty index [35]) has been widely applied, with minor variation, in research on older people, and been shown to have good construct, convergent, concurrent and predictive validity [36] (e.g., predicting mortality and risk of fractures, falls, hospitalisation, institutionalisation and visits to emergency departments). Our version of it comprised questions on unintended weight loss, exhaustion, poor grip strength (difficulty in opening jars), slow walking speed and low physical activity (in terms of regular activities sufficient to cause sweating).

Additionally, questions were posed on frequency of falls in the past 12 months (falls are associated with sarcopenia and frailty [37]); worsening of memory; regional pain making it difficult or impossible to get washed or dressed or do household chores (adapted from the widely used Nordic Questionnaire [38]); and difficulties in hearing (for which self-report of moderate to great difficulty in hearing conversation in a quiet room (or the wearing of a hearing aid) has been found, in a British national survey of hearing, to correspond to a mean hearing impairment of about 45 dB HL [39]).

#### Conditions of paid work

Among those in paid work, questions were asked about the type of employment, contract, employer’s size, length of employment, and entitlement to holiday, paid sick leave and an ill-health pension; also, about physical conditions of work and about the psychosocial work environment and feelings about work and retirement.

#### Physical working conditions

A series of questions was asked about exposures in an average working day to: kneeling/squatting (for >1 h/day in total), climbing a ladder, climbing stairs (>30 flights/day), digging/shovelling, lifting ≥10 kg by hand, hard physical work sufficient to cause sweating, and standing or walking (most of the day, >3 h at a time). Such exposures have been linked with a range of musculoskeletal disorders of interest, including osteoarthritis of the knee [40], meniscal injury [41] and back pain [42].

#### Psychological factors (including feelings about work)

Questions about psychosocial aspects of work were based broadly on the Karasek model of decision latitude, colleagues’ support, and work demands [43]. Additionally, questions were posed concerning job satisfaction (overall, and specifically with pay and working hours); sense of achievement gained through work; feeling appreciated by those at work, or unfairly criticised by them; difficult relationships and special friendships at work; lying awake at night worrying about work; and job insecurity in illness and in health. Subjects still in work were also asked about their retirement expectations (e.g., expected age at retirement, preferred age of retirement, expected pre-retirement changes in working hours).

### Social and financial circumstances

Affordability of retirement, family circumstances, outside interests, caring commitments and various other social and financial factors are liable to weigh in people’s retirement planning. The HEAF questionnaire will allow us to identify those who live alone or have no partner, those who have caring and non-paid voluntary commitments, or leisure pursuits, those who are the main household bread winners, and those who have others who are financially dependent on them. Questions on home ownership, on how well a person manages financially, and on the affordability of desired purchases provide a broad indication of current financial status; further questions concerned current and expected pension benefits and relative income in retirement.

### Follow-up

Subjects are being followed-up regularly, for five years initially, with a briefer postal questionnaire (Additional file 2). This is aimed at assessing changes from baseline in: job circumstances, with reasons (e.g., job loss, new job, job modification, for health-related or other reasons); health (e.g., changes in SRH, BSI, CES-D, MWBS, frailty, memory); and attitudes towards retirement (including those modified by spouse’s health and employment). Additional follow-up information on hospital referrals, new diagnoses, new treatments and new workplace injuries will come from CPRD files, by record linkage.

### CPRD-linked data

The CPRD records of participants offer a complementary source of information on health at baseline and follow-up. Taking musculoskeletal and mental health problems as examples: consultation episodes are classified by the hierarchical Read diagnostic coding system, enabling diagnoses to be defined broadly (e.g., Read code N: Musculoskeletal and connective tissue diseases; E: Mental disorders), in fine divisions of detail (e.g., N211: rotator cuff syndrome; N143: sciatica; N2165: prepatellar bursitis; E112: major depressive episode), and where relevant in functional or symptomatic terms (e.g., N3371 complex regional pain syndrome; N131: chronic/recurrent neck pain). Similarly, GPs’ prescriptions are logged using British National Formulary codes, from broad categories (e.g., 10.3: Drugs for the relief of soft-tissue inflammation) down to specific formulations, doses, and durations of treatment. In a separate scoping exercise, we have determined a suitable coding framework for consultations linked with injury that is likely to be occupational [44, 45].

Data linkage will focus on the following items from the CPRD record (in the 12 months prior to study entry and for the duration of follow-up):

- All hospital admissions, including all discharge diagnoses and procedures
- All GP consultations for musculoskeletal disorders (MSDs)
- All GP consultations for mental health problems
- All GP consultations for asthma or COPD
- All GP consultations for cardiovascular problems
- All GP consultations for diabetes and epilepsy
- All prescriptions related to these health problems (e.g., anxiolytics, hypnotics, sedatives, antidepressants, antipsychotics, narcotics, circulatory drugs, insulin, oral hypoglycaemics, antiepileptic medicines)
- All injuries likely to be occupational
- Frequency of GP consultations for all reasons combined
- Any records of height, weight, BMI, smoking habits, alcohol consumption.

### Plans for analysis

Analysis will consider health conditions as predictors of work outcome (e.g., the effect of MSDs on work capacity, employment status, and job retention); also, as the timing of events within the database is recorded for ill-health classified across a broad range of diagnoses, more complex causal chains can be examined such as the impact that MSD-related job loss might have on subsequent short-term mental health, or the impact of job loss or retention at older ages on mental health. Thus, with health and employment assessed at various time points (T_1_, T_2_,… T_n_) and denoted at each by H_1_, H_2_..H_n_ and E_1_, E_2_,…E_n_ respectively, or by change measures (ΔE_1–2_, ΔH_1–2_, etc.), with measures also of covariates of interest (C_1_, C_2_..) (Tables 3 and 4), we will assess: a) cross-sectional associations between (i) health and work status (H1 vs. E1), (ii) change in health and later work status/transition (ΔH_1–2_ vs. E_2_, ΔH_1–2_ vs. ΔE_1–2_), (iii) work transition and later health/change in health (ΔE_1–2_ vs. H_2_, ΔE_1–2_ vs. ΔH_1–2_); b) the longitudinal relation between (i) health (or health change) and work transition (H_1_ vs. ΔE_1–2_, ΔH_1–2_ vs. ΔE_2–3_ etc.), and between (ii) work transition and changed health (e.g., ΔE_1–2_ vs. ΔH_2–3_). Multi-level modelling will estimate effects with allowance for other personal and social factors as confounders or effect modifiers. Data will be combined across available time points using multi-level modelling to allow for non-independence of serial within-person measures. Modelling will adjust for covariates measured at the same time point (cross-sectional) or as in Table 4 (longitudinal). Analyses related to job transition will sub-classify by main reason for job change. More formally: let *H*_n_ = {H_1_, H_2_, …, H_n_} be the health history up to and including time n; let *E*_n_ = {E_1_, E_2_, …, E_n_} be the employment history up to and including time n; let *C*_n_ = {C_1_, C_2_, …, C_n_} be the effects of confounding up to and including time n. When H and E are binary, we will use multi-level logistic regression analysis to model logit (*H*_n_ = 1 | *H*_n-1_, *E*_n-1_, *C*_n-1_), where 2 ≤ n ≤ N_i,_ the number of observations of subject i. Such a model determines the extent to which all that is known at the beginning of an interval is associated with health at the end of the interval. One extension will be to include E_n_ and/or C_n_ as further predictors; another will be to run analyses in which the roles of health and employment are reversed. When H and E are continuous we will use multi-level linear regression analysis. Table 5 summarises certain health circumstances and the intended treatment of them. Analysis will also explore health and medication as predictors of occupational injury.

**Table 3:**
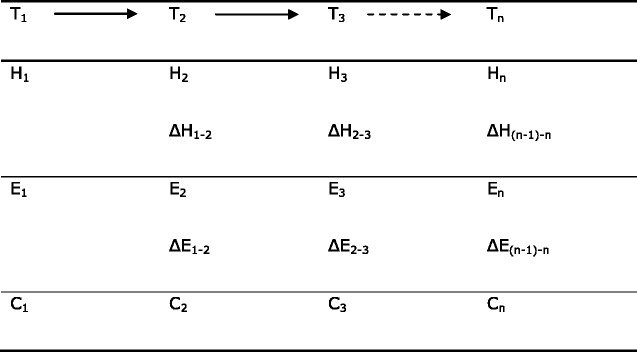
Serial observations of health status, employment status and other covariates

**Table 4:** Longitudinal analyses of time series data sets (for simplicity only 3 time points are presented)

Study question	Independent variable	Dependent variable	Covariate (s)
Effect of work on health			
Employment status as a predictor of health decline or improvement	E_1_	ΔH_1–2_	C_1_
Job change (e.g., new unemployment, planned retirement) as a predictor of health change	ΔE_1–2_	ΔH_2–3_	H_2_, C_2_
Longer term effects of employment status	E_1_	ΔH_2–4,_ ΔH_3–4_	H_2_, C_2_
	ΔE_1–2_	ΔH_2–4,_ ΔH_3–4_	
Effect of health on work			
Health as a predictor of job transition	H_1_	ΔE_1–2_	C_1_
Impact of health change on job status	ΔH_1–2_	ΔE_2–3_	E_2_, C_2_

**Table 5:** Some independent and dependent variables likely to feature in analysis (taking MSDs as an example)

a) Effect of health on work:	
Predictor variables	Outcome variables
Health or change in health	Employment status: unemployed, retired, ill-health retired, temporarily off sick, employed, other role (e.g., carer)
1) CPRD record: diagnosis (or treatment/worsening) of arthritis, soft tissue rheumatism, or other MSDs; or in those with MSDs, of concurrent… anxiety, depression, neurotic illness, insomnia, cardiovascular disease, new hospital treated illnesses etc.	Employment change: (new) involuntary job loss; planned normal retirement; early planned retirement; early ill-health retirement; re-employment
2) Questionnaire: in those with MSDs: change in pain symptoms, SRH, CES-D, BSI, sleep problems	
b) Effect of work on health:	
Predictor variables	Outcome variables
Employment status: unemployed, retired, ill-health retired, temporarily off sick, employed, other role (e.g., carer)	Change in health
	1) CPRD record: new diagnosis of, treatment for, worsening/recovery from … anxiety, depression, neurotic illness, insomnia, cardiovascular disease, hypertension; new hospital treated illnesses; altered frequency of GP visits
Employment change: (new) involuntary job loss; planned normal retirement; early planned retirement; early ill-health retirement; re-employment *(Including MSD-related employment changes)*	
	2) Questionnaire: (change in) … SRH, CES-D, BSI, sleep problems

### Sample size and study power

Power calculations were originally based on a target recruitment of 6000 at baseline which has since been exceeded (see below). On the original basis, assuming the employment rates in *Pension Trends 2012* [46], the disease frequencies in the *4th Morbidity Survey in General Practice* [47], 3 years of follow-up, with a 75 % response at follow-up and a 30 % rate of job loss, relative risks (RRs) of sickness-related job loss of 1.20 to 1.52 would be detectable across a range of common diseases (osteoarthritis, mental illness, chronic obstructive pulmonary disease, ischaemic heart disease) with an alpha 0.05 and a power 80 %. RRs of 1.54, 1.77, and 2.25 respectively would be detectable for new consultations with neurotic illness, hypertension and angina on the same basis. Actual study power should be higher, given the greater than anticipated numbers at baseline (see below).

## Response patterns at baseline

At baseline, the CPRD identified 40,357 individuals from the practices with qualifying dates of birth, but 997 of these subjects were excluded prior to mailing, principally on GPs’ advice (on grounds of terminal illness, recent bereavement, or de-registration). Of the 39,359 people who were approached to participate, 8,134 (20.7 %) returned a valid questionnaire, were in the target age range and consented to be followed up. Of these, some 200 agreed to receive further questionnaires but did not indicate whether we could access their anonymised NHS records. A further 1,291 completed a baseline return but did not agree to follow up, or did not offer their contact details.

Table 6 sets out the numbers of individuals excluded, approached and participating by deciles of relative deprivation. Classification of deprivation was based on the postcode details of their practice, and used the English Index of Multiple Deprivation 2010 (IMD 2010) [48]. This is a weighted average of 38 indicators in seven domains of deprivation – income, employment, health and disability, education skills and training, barriers to housing and social services, crime and living environment – calculated for each local area unit (Lower layer Super Output Area) in England. The sample included practices from all but one of the deciles of deprivation, but somewhat over-represented deciles 6 to 8 and under-represented deciles 1 to 3 – i.e., the sample was drawn from relatively more affluent catchment areas than the population of England as a whole. (The postcodes of individuals may have varied from that of their practice, but that information was available only for those who responded.) Response rates were lowest in the most deprived practices but otherwise varied relatively little by IMD 2010 grouping and showed no clear trend with index of deprivation.

**Table 6:** Recruitment and response rates at baseline by practice deprivation score

Decile of deprivation^a^	Practices (N)	Subjects				% of all participants
		No. excluded	No. approached	No. recruited	% recruited	
1 (worst)	2	77	2208	297	13.5 %	3.6 %
2	1	47	1830	322	17.6 %	4.0 %
3	0	0	0	0	0 %	0 %
4	1	66	1102	265	24.0 %	3.3 %
5	4	81	3944	673	17.1 %	8.3 %
6	5	389	11,940	2432	20.4 %	29.9 %
7	3	98	6080	1334	21.9 %	16.4 %
8	4	117	4761	1136	23.9 %	14.0 %
9	3	89	4879	1112	22.8 %	13.7 %
10 (best)	1	33	2615	563	21.5 %	6.9 %
All	24	997	39,359	8134	20.7 %	100.0 %

^a^IMD 2010 (see text)

Participating general practices were broadly spread geographically (Table 7), some 40 % of respondents being drawn from the South of England, 37 % from Central England, and 23 % from the North. Response rates were somewhat lower in London and the South East (16.1 %) than in other areas (19.5 to 22.0 %).

**Table 7:** Recruitment and response rates at baseline by location of practice

Location of practice	Practices (N)	Subjects			% of all participants
		No. approached	No. recruited	% recruited	
London/South East	3	3661	590	16.1 %	7.2 %
Central Southern	2	3635	745	20.5 %	9.2 %
South West	5	9003	1946	21.6 %	24.0 %
East	4	8438	1854	22.0 %	22.8 %
West Midlands	3	5843	1137	19.5 %	14.0 %
North East	4	6165	1344	21.8 %	16.5 %
North West	3	2614	518	19.8 %	6.4 %
All	24	39,359	8134	20.7 %	(100.0 %)

Response rates were higher at older ages and higher in women than men (Table 8). The overall sample, therefore, comprised more women than men (54 % vs. 46 %) and a greater proportion of individuals in the oldest age band (42 %) compared with the younger age bands (26–32 %). In comparison with the general population of England aged 50–64 years in June 2013, the sample was somewhat older.

**Table 8:** HEAF baseline response rates by age and sex

	N (%) responded	% of all	
		Sample^a^	Population^b^
Date of birth (approx. age at baseline^c^)			
1948–1922 (60–64)	3444 (28)	42	30
1953–1957 (55–59)	2582 (20)	32	32
1958–1962 (50–54)	2108 (14)	26	37
		(100)	(100)
Sex:			
Male	3707 (18)	46	49
Female	4427 (22)	54	51
		(100)	(100)

^a^column %

^b^Population of England aged 50–64 years, estimated for June 2013 by the Office for National Statistics (http://ons.gov.uk/ons/taxonomy/index.html?nscl=Population#tab-data-tables, accessed 12/3/15)

^c^based on the age when sampling lists were drawn up (a minority of subjects crossed age boundaries by the time of response – e.g., 557 were aged 65 years by then)

Table 9 summarises the work status of participants at baseline, by age and sex. In all, 5,509 respondents (68 %) were in paid work (employed or self-employed), the remainder being unemployed (7 %) or retired (26 %). These rates are very similar to those for all 50–64 year-olds in the UK, as judged by Labour Force Statistics for 2013 (67 % in paid work, 5 % unemployed, 29 % economically inactive) [49]. Some 5 % of HEAF participants held more than one paid job. Employment rates were lower in women than men, although differences by sex were not marked. However, they fell off steeply in the oldest band (46 % in work), among whom the retired proportion was substantial relative to 50–54 year-olds (51 % vs. 3 %).

**Table 9:** Employment status of respondents at baseline

Characteristic	Current work situation, N (%)				More than 1 current job, N (%)
	Employed	Self-employed	Unemployed	Retired	
Sex:					
Male	2099 (56.6)	593 (16.0)	230 (6.2)	785 (21.2)	169 (4.6)
Female	2475 (55.9)	342 (7.7)	306 (6.9)	1304 (29.5)	252 (5.7)
Age band (years):^a^					
50–54	1595 (75.7)	258 (12.2)	187 (8.9)	68 (3.2)	130 (6.2)
55–59	1752 (67.9)	322 (12.5)	233 (9.0)	275 (10.7)	179 (6.9)
60–64	1227 (35.6)	355 (10.3)	116 (3.4)	1746 (50.7)	112 (3.3)
All:	4574 (56.2)	935 (11.5)	536 (6.6)	2089 (25.7)	421 (5.2)

^a^A few were aged 64 years when sampling lists but 65 years at the time of response

The demographic characteristics of participants, overall and by sex, are given in Table 10. Subjects were typically Caucasian (98 %) and married or in a civil partnership (71 %), figures which compare with recent population values for England and Wales (of 93 % [50] and 70 % [51] respectively). One in seven had no qualifications, whereas a third had a university degree or a higher professional qualification; the comparative figures for adult residents of England and Wales were 23 % and 27 % respectively at the 2011 Census [52]. A high proportion of HEAF respondents were owner-occupiers, outright or with a mortgage (86 %), and this is above the average for home ownership in this age group across all of England and Wales (75 % in 2011) [53]. One in five lived alone. Descriptive information on respondents’ circumstances of work, finances, health, well-being, and retirement expectations and plans will feature in future reports.

**Table 10:** Demographic characteristics of respondents at baseline

Characteristic	Men N (%)	Women N (%)	All, N (%)
Ethnic group:			
Caucasian	3627 (98.1)	4334 (98.2)	7961 (98.2)
Other	70 (1.9)	78 (1.8)	148 (1.8)
Marital status:			
Married/civil partnership	2728 (73.8)	2995 (68.3)	5723 (70.8)
Widowed	83 (2.3)	242 (5.5)	325 (4.0)
Divorced	472 (12.8)	802 (18.3)	1274 (15.8)
Single	414 (11.2)	347 (7.9)	761 (9.4)
Educational qualification:^a^			
None	539 (14.5)	733 (16.6)	1272 (15.6)
School	631 (17.0)	1014 (22.9)	1645 (20.2)
Vocational training certificate	1203 (32.5)	1246 (28.2)	2449 (30.1)
University degree	631 (17.0)	652 (14.7)	1283 (15.8)
Higher professional qualification	703 (19.0)	782 (17.7)	1485 (18.3)
Home ownership:			
Owned outright	1849 (51.1)	2442 (56.7)	4291 (54.1)
Owned with a mortgage	1236 (34.2)	1259 (29.2)	2495 (31.5)
Rented	508 (14.0)	584 (14.0)	1092 (13.8)
Living rent free	26 (0.7)	22 (0.5)	48 (0.6)
Living alone:			
Yes	723 (19.8)	974 (22.3)	1697 (21.2)
No	2936 (80.2)	3386 (77.7)	6322 (78.8)

^a^highest attained level

At the time of writing, the stage 1 follow-up has been completed, with a response rate of some 80 %.

## Discussion

The HEAF study is set to generate substantial information which will be the subject of multiple reports.

A particular strength of the study is that it is nested within the dynamic population of patients registered with the CPRD; record linkage will thus allow cost-effective capture of detailed health data without reliance on the memory of study participants. The study involves a novel use of the CPRD database: prior studies have employed registry-based surveillance, nested case–control analyses and a randomised trial, but not so far observational follow-up of a cohort.

A limitation, at least at baseline, is that response rates were relatively low. However, the recruited sample, although somewhat older, better educated, and wealthier than 50–64 year-olds in the population at large, was reasonably representative, especially in terms of employment status, ethnicity and marital status, and included participants from most regions of England and most deciles of neighbourhood material affluence or deprivation. Moreover, in terms of the critical longitudinal questions, about work transitions and changes in health, the important response rates for judging internal validity will be those at follow-up (as explained in standard texts [54]), and these initially have been satisfactory. Also, while many variables of interest are self-reported (e.g., feelings about work and retirement), recall bias should be of less concern for subjective inquiries made prospectively, ahead of the main study outcomes.

## Conclusions

In summary, the HEAF study is a major new resource for the investigation of health risks and benefits of extended working lives. Although the data collected have inevitable limitations, they should allow exploration of many policy-relevant questions on work at older ages and the factors that may support the continuing employment, healthy ageing, and well-being of the ageing workforce. Opportunities exist, also, to make face-to-face objective assessments of physical and cognitive function within subsamples of the HEAF cohort, and to pool data with similarly aimed cohort studies of work transitions in older people from other countries, thereby adding to the cohort’s long-term value.

## Additional files

Additional file 1:
**Baseline HEAF Questionnaire.** (PDF 1087 kb) Additional file 2:
**HEAF Follow Up Questionnaire.** (PDF 450 kb)
