# Supplementary material for: Health and Employment after Fifty (HEAF): a new prospective cohort study
Source: BMC Public Health. 2015 Oct 19;15:1071. doi: 10.1186/s12889-015-2396-8 (PMC4615332; doi:10.1186/s12889-015-2396-8)
Supplement: Additional file 1: — Baseline HEAF Questionnaire. (PDF 1087 kb) [file 12889_2015_2396_MOESM1_ESM.pdf]

FORM A SERIAL NO:

Lifecourse Epidemiology Unit

MRC

MEDICAL RESEARCH COUNCIL

UNIVERSITY OF Southampton

Health & Employment After Fifty (HEAF Study)

The answers given on this form are confidential.
Replies will only been seen by a small medical research team

Section One: About Yourself

Please fill in today's date

Day: ☐☐ Month: ☐☐ Year: 2 0 1 ☐

1. Please fill in your date of birth

Day: ☐☐ Month: ☐☐ Year: ☐☐☐☐

2. And your sex

Male ☐ Female ☐

3. Please indicate your ethnic origin *(Tick one box)*

a) White ☐ b) Black-Caribbean ☐ c) Black-African ☐

d) Black-Other ☐ e) Indian ☐ f) Pakistani ☐

g) Bangladeshi ☐ h) Chinese ☐ i) Other *(please specify)* ☐

----

4. What is your current marital status? *(Tick one box)*

a) Married ☐ b) Single ☐ c) Civil partnership ☐

d) Widowed ☐ e) Divorced ☐

5. At what age did you leave school?

☐☐ Years old

6. Did you do any further education or training after school? *(Tick all the boxes that apply)*

a) Apprenticeship ☐

b) Full-time College or University course ☐

c) Part-time College or University course (including day release or night classes) ☐

d) Other *(please specify)* ☐

----

7. Do you have any of the following qualifications? *(Tick all the boxes that apply)*

a) O Levels/GCSEs (or equivalents) ☐

b) A Levels (or equivalents) ☐

c) Vocational training certificate(s) (e.g. City and Guilds, NVQ) ☐

d) University degree(s) or HND ☐

e) Higher professional qualifications (e.g. in accountancy, law, etc) ☐

Section One: About Yourself *(continued)*

8. In an average week, roughly how many hours would you spend doing the following activities? *(Please answer each question)*

		Hours per week
a)	Working in a paid job	
b)	Doing housework in your own home or for your family	
c)	Doing DIY jobs in your own home or for your family	
d)	Giving personal care to someone in your home or family	
e)	Working in an unpaid job for others outside your home and family (e.g. as a volunteer for a charity)	
f)	Playing sports	
g)	Doing hobbies (other than sports and DIY in the home)	
h)	Watching television	
i)	Reading for pleasure	
j)	Other leisure activities	

9. In an average week, and outside any paid jobs that you do, roughly how many hours would you spend doing the following activities? *(Please answer each question)*

		Hours per week
a)	Physical activities sufficient to make you hot or sweaty (e.g. heavy gardening, dancing, cycling, jogging)	
b)	Meeting or doing things with friends or relatives who do not live in your home	

Section Two: Employment

10. Which of the following best describes your present work situation? *(Tick one box)*

a) Employed ☐ b) Self-employed ☐

c) Unemployed ☐ d) Retired ☐

If you currently have a paid job, please go to ***Section 3****, beginning at* ***Question 16*** *on* ***page 4.***

If you do not currently have a paid job, please continue with ***Question 11***

11. Have you ever had a paid job? *(Tick one box)*

a) Yes ☐ b) No ☐

If you have never had a paid job, please go to ***Section 5,*** *beginning at* ***Question 49*** *on* ***page 9****.*

If you have had a paid job in the past, please continue with ***Question 12****.*

12. What was your last paid job?

Occupation: ______________________________

13. When did you leave this job?

☐☐ ☐☐☐☐

Month **Year**

14. Did you leave because of a health problem? *(Tick one box)*

a) No, not at all ☐

b) Yes, a health problem was **the main** reason for leaving ☐

c) Yes, a health problem was **part of** the reason for leaving ☐

15. If there was a health problem, what type of problem was it? (*Tick all the boxes that apply*)

a) A problem with your back, neck, arm, shoulder or leg ☐

b) A mental health problem or stress ☐

c) A problem with your heart or lungs ☐

d) Another type of health problem ☐

e) Not applicable, no health problem ☐

Section Three: Your Main Paid Job

Please answer this section only if you currently have a paid job.
If you do not have a paid job please go to Section Five, Question 49 on page 9.

The next few questions are about your MAIN paid job

16. What is your MAIN occupation at the moment?

a) Occupation *(e.g. secretary, teacher, builder)* --

and in what industry do you work?

b) Industry (e.g. farming, shipyard, car factory, shoe shop, hospital, insurance office) --

17. If you added up all the time you have been doing this kind of work, roughly how long would that make altogether?

a) Less than 1 year ☐ b) 1 to 5 years ☐ c) More than 5 years ☐

18. Is your contract of employment permanent or temporary/renewable?

a) Permanent ☐ b) Temporary/renewable ☐ c) Not applicable (self-employed) ☐

19. How long in total have you worked for your present employer?
(If self employed, how long in total have you worked for yourself?)

a) Less than 1 year ☐ b) 1 to 5 years ☐ c) More than 5 years ☐

20. Roughly how many people in total work for your employer?
(If self-employed, please indicate the number of people in total you employ)

a) Just you ☐ b) 2 – 9 ☐ c) 10 – 29 ☐

d) 30 – 499 ☐ e) 500 or more ☐

21. Does your main job involve rotating or variable shifts?

a) Often ☐ b) Sometimes ☐ c) Rarely/never ☐

22. Does your main job involve night work (i.e. between 2.00 a.m. and 4.00 a.m.)?

a) Often ☐ b) Sometimes ☐ c) Rarely/never ☐

Section Three: Your Main Paid Job *(continued)*

23. In your main job, does an average day at work involve any of the following activities?
(Please tick yes or no for each activity)

		Yes	No
a)	Kneeling or squatting for longer than 1 hour per day in total	☐	☐
b)	Climbing a ladder	☐	☐
c)	Climbing up and down more than 30 flights of stairs per day	☐	☐
d)	Digging or shovelling	☐	☐
e)	Lifting weights of 10 kg (25 lbs) or more by hand	☐	☐
f)	Standing or walking for most of the day	☐	☐
g)	Standing or walking for more than 3 hours at a time	☐	☐
h)	Hard physical work that makes you hot or sweaty	☐	☐

24. Is driving part of your main job?
(Tick one box. NB This does not include travel to or from your main place of work)

a) Essential to the job ☐ b) A part of the job, but not essential ☐ c) No ☐

25. Ignoring overtime, does your main job give you a fixed salary, or are you paid according to your output (e.g. the number of tasks you do or things you make)? *(Tick one box)*

a) Fixed salary ☐ b) Paid by output ☐

26. In your main job, do you have a choice in deciding what you do, how you do things, or when you do things? *(Tick one box)*

a) Often ☐ b) Sometimes ☐ c) Rarely/never ☐

27. Do you have a fixed time when you <u>have to</u> begin work? *(Tick one box)*

a) All work days ☐ b) Most work days ☐ c) Some work days ☐

d) Never (I choose for myself) ☐

28. How much holiday are you allowed from your job per year (including Bank Holidays)?
*(Answer a, **or** b)*

a) ☐☐ Days or b) ☐ No fixed limit *(Please tick)*

Section Three: Your Main Paid Job *(continued)*

29. How much holiday do you take each year in your job (including Bank Holidays)?

☐☐ days

30. When you have difficulties at work, how often do you get help and support from your colleagues, supervisor or manager? *(Tick one box)*

a) Often ☐ b) Sometimes ☐ c) Rarely/never ☐

d) Not applicable (work alone) ☐

31. Do you ever lie awake at night worrying about work or angry about work? *(Tick one box)*

a) Often ☐ b) Sometimes ☐ c) Rarely/never ☐

32. How satisfied are you with the amount you are <u>paid</u> in your job, all things considered? *(Tick one box)*

a) Very satisfied ☐ b) Satisfied/fairly satisfied ☐

c) Dissatisfied ☐ d) Very dissatisfied ☐

33. How satisfied are you with your <u>working hours and your work timetable</u> (e.g. start and finish time), all things considered? *(Tick one box)*

a) Very satisfied ☐ b) Satisfied/fairly satisfied ☐

c) Dissatisfied ☐ d) Very dissatisfied ☐

34. Does your work give you a feeling of achievement? *(Tick one box)*

a) Often ☐ b) Sometimes ☐ c) Rarely/never ☐

35. In your work, do you feel appreciated by others (managers, colleagues, customers etc)? *(Tick one box)*

a) Often ☐ b) Sometimes ☐ c) Rarely/never ☐

36. Do you have friends at work with whom you also spend time outside work? *(Tick one box)*

a) Yes ☐ b) No ☐

Section Three: Your Main Paid Job *(continued)*

37. Is there anyone at work you find very difficult to get on with? *(Tick one box)*

a) Yes ☐ b) No ☐

38. Do you ever get criticised unfairly at work? *(Tick one box)*

a) Often ☐ b) Sometimes ☐ c) Rarely/never ☐

39. How satisfied have you been with your job as a whole, taking everything into consideration? *(Tick one box)*

a) Very satisfied ☐ b) Satisfied/fairly satisfied ☐

c) Dissatisfied ☐ d) Very dissatisfied ☐

40. Provided that you stay well, how secure do you feel your job is? *(Tick one box)*

a) Very secure ☐ b) Secure ☐

c) Rather insecure ☐ d) Very insecure ☐

41. How secure do you feel your job would be if you had an illness that kept you off work for three months or more? *(Tick one box)*

a) Very secure ☐ b) Secure ☐

c) Rather insecure ☐ d) Very insecure ☐

42. If you fell ill and were off work, how long could you get your normal full pay (excluding bonuses)? *(Tick one box)*

a) Less than one week ☐ b) 1 to 4 weeks ☐ c) 1 to 6 months ☐

d) More than 6 months ☐ e) Not sure ☐

43. If you had a long-term health problem, might you qualify for an ill-health retirement pension (from your employer or insurance)? *(Tick one box)*

a) Yes ☐ b) No ☐ c) Don't know ☐

44. Currently, how well do you cope with the physical demands of your job? *(Tick one box)*

a) Easily ☐ b) With some difficulty ☐

c) With great difficulty ☐ d) Not coping ☐

Section Three: Your Main Paid Job *(continued)*

45. Currently, how well do you cope with the mental demands of your job? *(Tick one box)*

a) Easily ☐ b) With some difficulty ☐

c) With great difficulty ☐ d) Not coping ☐

46. Do you expect that you will still be able (physically and mentally) to carry out the same kind of work in two years time? *(Tick one box)*

a) Yes ☐ b) No ☐ c) Not sure ☐

Section Four: Other Paid Jobs

The last section concerned your MAIN job. This section concerns any other PAID jobs that you may have.

47. Do you have any PAID jobs, other than the one you told us about in the last section? *(Tick one box)*

a) Yes ☐ b) No ☐

*If **no**, please go to **Section Five, page 9**.* *If **yes**, please list the other paid job(s) you do and the average working hours per week*

48. OCCUPATION	Hours per week
a)	
b)	
c)	
d)	
e)	

Section Five: Personal Finance

49. How many adults (including yourself) live in your household? ☐☐

50. And how many children under 18 years old? ☐☐

51. Roughly how much of the total household income comes from money which you personally earn in a paid job?
(Please do not include any money that you receive from pensions or investments) (Tick one box)

a) None ☐ b) Less than a quarter ☐ c) Between a quarter and a half ☐

d) Half or more ☐

52. Is anyone outside your household financially dependent on you? *(Tick one box)*

a) Yes ☐ b) No ☐

53. Is your home.... *(Tick the box that best applies)*

a) Owned outright by you or someone else in the household? ☐

b) Owned by you or someone else in the household, but with a mortgage? ☐

c) Rented? ☐

d) Rent free? ☐

e) Other? *(please specify)* ☐

----

54. How well do you feel you are managing financially these days? *(Tick the box that best applies)*

a) Living comfortably ☐

b) Doing alright ☐

c) Just about getting by ☐

d) Finding it difficult to make ends meet ☐

e) Finding it very difficult to make ends meet ☐

55. Are there things which you used to have, and which you would like to have now, but can no longer afford? *(Tick one box)*

a) No ☐ b) A few things ☐ c) Many things ☐

56. Are there things which your friends or family have, that you would like to have but cannot afford? *(Tick one box)*

a) No ☐ b) A few things ☐ c) Many things ☐

Section Five: Personal Finance *(continued)*

57. Apart from any state pension, do you currently receive a private or employers' pension? *(Tick one box)*

a) No ☐ b) Yes ☐

58. If yes, do you receive an employers' ill health pension? *(Tick one box)*

a) No ☐ b) Yes ☐ c) Not applicable ☐
(do not receive an employers' pension)

59. Do you expect to receive a private or employer's pension in the future (in addition to any pension that you already get)? *(Tick one box)*

a) No ☐ b) Yes ☐

60. If yes, from what age would this/these private or employers' pension(s) be paid?
(Please fill in more than one set of boxes if you are due to get several pensions at different ages)

a) ☐☐ years b) ☐☐ years c) ☐☐ years

61. If you are already fully retired, please tick this box and move to Section 6, page 11. Otherwise please continue with question 62. ☐

62. When you are fully retired and receiving any pensions that are due to you, how will your total personal income compare with what you get now? *(Tick one box)*

a) Less than a quarter ☐ b) Between a quarter and a half ☐ c) Half or more ☐

63. At what age do you expect to retire fully?

a) ☐☐ years old

64. Do you expect to reduce your paid work before you retire fully? (e.g. by working shorter hours for less pay)? *(Tick one box)*

a) No ☐ b) Yes ☐ c) Not sure ☐

65. In an ideal world, at what age would you like to retire fully?

a) ☐☐ years old or b) never *(Please tick)* ☐

Section Six: Health

66. In general would you say your health is? *(Tick one box)*

a) Excellent ☐ b) Very good ☐ c) Good ☐ d) Fair ☐ e) Poor ☐

67. Please give your height and your weight

Height ☐ ft ☐☐ ins or ☐☐☐ cm

Weight ☐☐ st ☐☐ lbs or ☐☐☐ kg

68. Thinking about your weight, in the past 12 months have you lost more than 10 pounds (4.5 kg) *unintentionally (i.e. without dieting or exercise)?*

a) Yes ☐ b) No ☐

69. How much of the following do you drink per week, on average?

a) Beer, cider, lager ☐☐ Pints b) Wine, sherry ☐☐ Glasses

c) Spirits, liqueurs ☐☐ Measures

70. Have you ever smoked regularly *(at least once a day for a month or longer)***?**

a) No ☐ b) Yes ☐ *(If no, go to Question 74)*

71. If yes, how old were you when you first smoked regularly? ☐☐ years old

72. Do you still smoke regularly? *(Tick one box)*

a) No ☐ b) Yes ☐ *(If yes, go to Question 74)*

73. If No, how old were you when you last smoked regularly? ☐☐ years old

74. Do you have difficulty with any of the following activities? *(One tick for each row)*

		No problem	Mild Problem	Moderate Problem	Severe Problem
a)	Walking	☐	☐	☐	☐
b)	Getting up from sitting	☐	☐	☐	☐
c)	Opening jars that have never been opened	☐	☐	☐	☐

Section Six: Health *(continued)*

75. Have you ever had any of the following operations? *(If yes, give the age when you first had the operation)*

a) Hip replacement No ☐ Yes ☐ Age ☐☐ years

b) Knee replacement No ☐ Yes ☐ Age ☐☐ years

c) Knee cartilage surgery No ☐ Yes ☐ Age ☐☐ years

76. Which of the following best describes your walking speed? *(Tick one box)*

a) Unable to walk ☐ b) Very slow ☐ c) Stroll at an easy pace ☐

d) Normal pace ☐ e) Fairly brisk ☐ f) Fast ☐

77. Have you had any falls in the past 12 months? *(Tick one box)*

a) No falls ☐ b) One fall ☐ c) More than one fall ☐

78. Do you have problems with your memory? *(Tick one box)*

a) No problems ☐ b) Sometimes, but not a serious problem ☐ c) serious problems ☐

79. Do you think your memory has got worse over the past 2 years? *(Tick one box)*

a) No ☐ b) A bit worse ☐ c) A lot worse ☐

80. Do you wear a hearing aid?

No ☐ Yes ☐ *(if Yes, please answer the next question (Q81) assuming that you are <u>not</u> wearing the aid at the time).*

81. How well can you hear a person who is talking to you in a quiet room?

a) With no or slight difficulty ☐ b) With moderate difficulty ☐ c) With great difficulty or not at all ☐

82. Below is a list of <u>problems that people sometimes have</u>. Please read each one carefully and tick the box that best describes how much that problem has distressed or bothered you during the <u>past 7 days</u> including today *(One tick for each row)*

		Not at all	A little bit	Moderately	Quite a bit	Extremely
a)	Faintness or dizziness	☐	☐	☐	☐	☐
b)	Pains in the heart or chest	☐	☐	☐	☐	☐
c)	Nausea or upset stomach	☐	☐	☐	☐	☐
d)	Trouble getting your breath	☐	☐	☐	☐	☐
e)	Hot or cold spells	☐	☐	☐	☐	☐

83. Below is a list of ways you might have felt or behaved – please tell us how often you have felt this way during the past 7 days including today *(One tick for each row)*

		During the past 7 days			
		Rarely or none of the time (less than one day)	Some or a little of the time (1-2 days)	Occasionally or a moderate amount of the time (3-4 days)	Most or all of the time (5-7 days)
a)	I was bothered by things that usually didn't bother me	☐	☐	☐	☐
b)	I did not feel like eating; my appetite was poor	☐	☐	☐	☐
c)	I felt that I could not shake off feeling low, even with help from my family and/or friends	☐	☐	☐	☐
d)	I felt I was just as good as other people	☐	☐	☐	☐
e)	I had trouble keeping my mind on what I was doing	☐	☐	☐	☐
f)	I felt depressed	☐	☐	☐	☐
g)	I felt that everything I did was an effort	☐	☐	☐	☐
h)	I felt hopeful about the future	☐	☐	☐	☐
i)	I thought my life had been a failure	☐	☐	☐	☐
j)	I felt fearful	☐	☐	☐	☐
k)	My sleep was restless	☐	☐	☐	☐
l)	I was happy	☐	☐	☐	☐
m)	I talked less than usual	☐	☐	☐	☐
n)	I felt lonely	☐	☐	☐	☐
o)	People were unfriendly	☐	☐	☐	☐
p)	I enjoyed life	☐	☐	☐	☐
q)	I had crying spells	☐	☐	☐	☐
r)	I felt sad	☐	☐	☐	☐
s)	I felt that people dislike me	☐	☐	☐	☐
t)	I could not get "going"	☐	☐	☐	☐

84. Below are some statements about feelings and thoughts. Please tick the box in each row that best describes your experience of each over the last 2 weeks *(One tick for each row)*

		None of the time	Rarely	Some of the time	Often	All of the time
a)	I've been feeling optimistic about the future	☐	☐	☐	☐	☐
b)	I've been feeling useful	☐	☐	☐	☐	☐
c)	I've been feeling relaxed	☐	☐	☐	☐	☐
d)	I've been feeling interested in other people	☐	☐	☐	☐	☐
e)	I've had energy to spare	☐	☐	☐	☐	☐
f)	I've been dealing with problems well	☐	☐	☐	☐	☐
g)	I've been thinking clearly	☐	☐	☐	☐	☐
h)	I've been feeling good about myself	☐	☐	☐	☐	☐
i)	I've been feeling close to other people	☐	☐	☐	☐	☐
j)	I've been feeling confident	☐	☐	☐	☐	☐
k)	I've been able to make up my own mind about things	☐	☐	☐	☐	☐
l)	I've been feeling loved	☐	☐	☐	☐	☐
m)	I've been interested in new things	☐	☐	☐	☐	☐
n)	I've been feeling cheerful	☐	☐	☐	☐	☐

85. How much have you been troubled by sleep problems in the past 3 months? *(One tick for each row)*

		No problem	Mild Problem	Moderate Problem	Severe Problem
a)	Difficulty falling asleep	☐	☐	☐	☐
b)	Difficulty staying asleep	☐	☐	☐	☐
c)	Waking up too early	☐	☐	☐	☐
d)	Not feeling refreshed in the morning	☐	☐	☐	☐

Section Six: Health *(continued)*

Past 12 months

86. During the <u>past 12 months</u>, have you had pain in your BACK or NECK for a month or longer that made it difficult or impossible to get washed or dressed or do household chores?

a) No ☐ b) Yes ☐

87. During the <u>past 12 months</u>, have you had pain in your ARM(S) or SHOULDER(S) for a month or longer that made it difficult or impossible to get washed or dressed or do household chores?

a) No ☐ b) Yes ☐

88. During the <u>past 12 months</u>, have you had pain in your LEG(S) for a month or longer that made it difficult or impossible to get washed or dressed or do household chores?

a) No ☐ b) Yes ☐

89. During the <u>past 12 months</u>, how many days have you had off work in total because of problems with your health? *(Tick one box)*

a) No time ☐ b) Less than 5 days ☐ c) 5 to 20 days ☐

d) More than 20 days ☐ e) Not applicable *(not working over this time)* ☐

90. During the <u>past 12 months</u>, how many days have you had off work in total because of pain in your back, neck, arms, shoulders or legs? *(Tick one box)*

a) No time ☐ b) Less than 5 days ☐ c) 5 to 20 days ☐

d) More than 20 days ☐ e) Not applicable *(not working over this time)* ☐

91. During the <u>past 12 months</u>, have you had to cut down, avoid or change what you normally do at work because of health problems? *(Tick one box)*

a) Yes, a lot ☐ b) Yes, a little ☐ c) No, not at all ☐

d) Not applicable *(not working over this time)* ☐

You have finished FORM A. Please place this form in the pre-paid envelope supplied.

Please also complete FORM B, add it to the prepaid envelope, and post both forms back

THANK YOU!
