# Supplementary material for: Health and Employment after Fifty (HEAF): a new prospective cohort study
Source: BMC Public Health. 2015 Oct 19;15:1071. doi: 10.1186/s12889-015-2396-8 (PMC4615332; doi:10.1186/s12889-015-2396-8)
Supplement: Additional file 2: — HEAF Follow Up Questionnaire. (PDF 450 kb) [file 12889_2015_2396_MOESM2_ESM.pdf]

SERIAL NO:

MEDICAL RESEARCH COUNCIL

Health & Employment After Fifty (HEAF Study): Follow-up Questionnaire

The answers given on this form are confidential.
Replies will only been seen by a small medical research team

Section One: About Yourself and Your Work

Please fill in today's date

Day	Month	Year
		2 0 1

1. Please fill in your date of birth

Day	Month	Year

2. What is your current marital status? *(Tick one box)*

a) Married ☐ b) Single ☐ c) Civil partnership ☐

d) Widowed ☐ e) Divorced ☐ f) Living with a partner ☐

3. Which of the following best describes your present work situation? *(Tick one box)*

a) Employed ☐ b) Self-employed ☐

c) Unemployed ☐ d) Retired ☐

4. Has your employment position changed since we last contacted you about a year ago? *(Please tick the box that best applies to you).*

I did not have a paid job when you last contacted me, and I do not have a paid job now ☐
*(Please go to **Section 2** on **page 8**, starting at **Question 45**)*

I have the same main job as when you last contacted me ☐
*(Please go to **Question 40** on **page 7**)*

My employment position has changed since you last contacted me. ☐
*(Please continue with **Question 5**)*

The following questions are only for those whose employment position has changed since we last contacted them.

5. In the time since we last contacted you, have you left the main job you were doing at that time?

No, I did not have a job when lasted contacted. ☐
*(Please go to **Question 9** on **page 2**)*

Yes ☐
*(Please continue with **Question 6**)*

6. When did you leave the job?

Month	Year

7. Did you leave because of a health problem? *(Tick one box)*

a) No, not at all ☐

b) Yes, a health problem was **the main** reason for leaving ☐

c) Yes, a health problem was **part of** the reason for leaving ☐

8. If there was a health problem, what type of problem was it? *(Tick all the boxes that apply)*

a) A problem with your back, neck, arm, shoulder or leg ☐

b) A mental health problem or stress ☐

c) A problem with your heart or lungs ☐

d) Another type of health problem ☐

e) Not applicable, no health problem ☐

9. Do you currently have a paid job?

a) No ☐ *(Please go to **Section 2** on **page 8** starting at **Question 45**)*

b) Yes ☐ *(Please continue with **Question 10**)*

10. What is your MAIN occupation at the moment?

a) Occupation (e.g. secretary, teacher, builder) ....

and in what industry do you work?

b) Industry (e.g. farming, shipyard, car factory, shoe shop, hospital, insurance office) ....

11. When did you start this job?

☐☐ Month ☐☐☐☐ Year

12. Is your contract of employment permanent or temporary/renewable?

a) Permanent ☐

b) Temporary/renewable ☐

c) Not applicable (self-employed) ☐

Section One: About Yourself and Your Work

13. Roughly how many people in total work for your employer?
(If self-employed, please indicate the number of people in total you employ)

a) Just you ☐ b) 2 – 9 ☐ c) 10 – 29 ☐

d) 30 – 499 ☐ e) 500 or more ☐

14. Does your main job involve rotating or variable shifts?

a) Often ☐ b) Sometimes ☐ c) Rarely/never ☐

15. Does your main job involve night work (i.e. between 2.00 a.m. and 4.00 a.m.)?

a) Often ☐ b) Sometimes ☐ c) Rarely/never ☐

16. In your main job, does an average day at work involve any of the following activities?
(Please tick yes or no for each activity)

		Yes	No
a)	Kneeling or squatting for longer than 1 hour per day in total	☐	☐
b)	Climbing a ladder	☐	☐
c)	Climbing up and down more than 30 flights of stairs per day	☐	☐
d)	Digging or shovelling	☐	☐
e)	Lifting weights of 10 kg (25 lbs) or more by hand	☐	☐
f)	Standing or walking for most of the day	☐	☐
g)	Standing or walking for more than 3 hours at a time	☐	☐
h)	Hard physical work that makes you hot or sweaty	☐	☐

17. Is driving part of your main job?
(Tick one box. NB This does not include travel to or from your main place of work)

a) Essential to the job ☐ b) A part of the job, but not essential ☐ c) No ☐

Section One: About Yourself and Your Work

18. Ignoring overtime, does your main job give you a fixed salary, or are you paid according to your output (e.g. the number of tasks you do or things you make)? *(Tick one box)*

a) Fixed salary ☐ b) Paid by output ☐

19. In your main job, do you have a choice in deciding what you do, how you do things, or when you do things? *(Tick one box)*

a) Often ☐ b) Sometimes ☐ c) Rarely/never ☐

20. Do you have a fixed time when you have to begin work? *(Tick one box)*

a) All work days ☐ b) Most work days ☐ c) Some work days ☐

d) Never (I choose for myself) ☐

21. How much holiday are you allowed from your job per year (including Bank Holidays)? *(Answer a,* ***or*** *b)*

a) ☐☐ Days or b) ☐ No fixed limit *(Please tick)*

22. How much holiday do you take each year in your job (including Bank Holidays)?

☐☐ days

23. When you have difficulties at work, how often do you get help and support from your colleagues, supervisor or manager? *(Tick one box)*

a) Often ☐ b) Sometimes ☐ c) Rarely/never ☐

d) Not applicable (work alone) ☐

24. Do you ever lie awake at night worrying about work or angry about work*? (Tick one box)*

a) Often ☐ b) Sometimes ☐ c) Rarely/never ☐

25. How satisfied are you with the amount you are paid in your job, all things considered? *(Tick one box)*

a) Very satisfied ☐ b) Satisfied/fairly satisfied ☐

c) Dissatisfied ☐ d) Very dissatisfied ☐

Section One: About Yourself and Your Work

26. How satisfied are you with your working hours and your work timetable (e.g. start and finish time), all things considered? *(Tick one box)*

a) Very satisfied ☐ b) Satisfied/fairly satisfied ☐

c) Dissatisfied ☐ d) Very dissatisfied ☐

27. Does your work give you a feeling of achievement? *(Tick one box)*

a) Often ☐ b) Sometimes ☐ c) Rarely/never ☐

28. In your work, do you feel appreciated by others (managers, colleagues, customers etc)? *(Tick one box)*

a) Often ☐ b) Sometimes ☐ c) Rarely/never ☐

29. Do you have friends at work with whom you also spend time outside work? *(Tick one box)*

a) Yes ☐ b) No ☐

30. Is there anyone at work you find very difficult to get on with? *(Tick one box)*

a) Yes ☐ b) No ☐

31. Do you ever get criticised unfairly at work? *(Tick one box)*

a) Often ☐ b) Sometimes ☐ c) Rarely/never ☐

32. How satisfied have you been with your job as a whole, taking everything into consideration? *(Tick one box)*

a) Very satisfied ☐ b) Satisfied/fairly satisfied ☐

c) Dissatisfied ☐ d) Very dissatisfied ☐

33. Provided that you stay well, how secure do you feel your job is? *(Tick one box)*

a) Very secure ☐ b) Secure ☐

c) Rather insecure ☐ d) Very insecure ☐

Section One: About Yourself and Your Work

34. How secure do you feel your job would be if you had an illness that kept you off work for three months or more? *(Tick one box)*

a) Very secure ☐ b) Secure ☐

c) Rather insecure ☐ d) Very insecure ☐

35. If you fell ill and were off work, how long could you get your normal full pay (excluding bonuses)? *(Tick one box)*

a) Less than one week ☐ b) 1 to 4 weeks ☐ c) 1 to 6 months ☐

d) More than 6 months ☐ e) Not sure ☐

36. If you had a long-term health problem, might you qualify for an ill-health retirement pension (from your employer or insurance)? *(Tick one box)*

a) Yes ☐ b) No ☐ c) Don't know ☐

37. Currently, how well do you cope with the physical demands of your job? *(Tick one box)*

a) Easily ☐ b) Just about ☐ c) With some difficulty ☐

d) With great difficulty ☐ e) Not coping ☐

38. Currently, how well do you cope with the mental demands of your job? *(Tick one box)*

a) Easily ☐ b) Just about ☐ c) With some difficulty ☐

d) With great difficulty ☐ e) Not coping ☐

39. Do you expect that you will still be able (physically and mentally) to carry out the same kind of work in two years time? *(Tick one box)*

a) Yes ☐ b) No ☐ c) Not sure ☐

Section One: About Yourself and Your Work

Questions 40 – 44 are only for those who are still in the same main job as when last contacted. ***If this does not apply to you, please move to Section 2 on page 8 starting with Question 45.***

40. Is your main job more or less the same as when we last contacted you (i.e. hours worked, tasks involved, support from managers and colleagues)?

a) No ☐ b) Yes ☐ *If yes, please move on to* ***Question 42***

41. If no, how has your job changed since we last contacted you?

i) How has it changed? ______________________________

____

____

ii) Have you reduced or changed what you do at work because of a health problem?

No ☐

Yes ☐ *Please describe the health problems* ______________________________

____

____

42. Currently, how well do you cope with the physical demands of your job? *(Tick one box)*

a) Easily ☐ b) With some difficulty ☐ c) With great difficulty ☐

d) Not coping ☐

43. Currently, how well do you cope with the mental demands of your job? *(Tick one box)*

a) Easily ☐ b) With some difficulty ☐ c) With great difficulty ☐

d) Not coping ☐

44. Do you expect that you will still be able (physically and mentally) to carry out the same kind of work in two years time? *(Tick one box)*

a) Yes ☐ b) No ☐ c) Not sure ☐

Section Two: Personal Finance

45. How well do you feel you are managing financially these days? *(Tick the box that best applies)*

a) Living comfortably ☐
b) Doing alright ☐
c) Just about getting by ☐
d) Finding it difficult to make ends meet ☐
e) Finding it very difficult to make ends meet ☐

46. Are there things which you used to have, and which you would like to have now, but can no longer afford? *(Tick one box)*

a) No ☐ b) A few things ☐ c) Many things ☐

47. Are there things which your friends or family have, that you would like to have but cannot afford? *(Tick one box)*

a) No ☐ b) A few things ☐ c) Many things ☐

48. Are you currently receiving an ill-health retirement pension?

a) No ☐ b) Yes ☐

49. If you are already fully retired, please tick this box and move to Section 3 on page 9, starting at Question 53. *(Otherwise, please continue with question 50).* ☐

50. At what age do you expect to retire fully?

a) ☐☐ years old

51. Do you expect to reduce your paid work before you retire fully? (e.g. by working shorter hours for less pay) *(Tick one box)*

a) No ☐ b) Yes ☐ c) Not sure ☐

52. In an ideal world, at what age would you like to retire fully?

a) ☐☐ years old or never ☐

Section Three: Health

53. In general would you say your health is? *(Tick one box)*

a) Excellent ☐ b) Very good ☐ c) Good ☐ d) Fair ☐ e) Poor ☐

54. How much of the following do you drink per week, on average?

a) Beer, cider, lager ☐☐ Pints b) Wine, sherry ☐☐ Glasses c) Spirits, Liqueurs ☐☐ measures

55. Below are some statements about feelings and thoughts. Please tick the box in each row that best describes your experience of each over the last __2 weeks__ *(One tick for each row)*

	None of the time	Rarely	Some of the time	Often	All of the time
a) I've been feeling optimistic about the future	☐	☐	☐	☐	☐
b) I've been feeling useful	☐	☐	☐	☐	☐
c) I've been feeling relaxed	☐	☐	☐	☐	☐
d) I've been feeling interested in other people	☐	☐	☐	☐	☐
e) I've had energy to spare	☐	☐	☐	☐	☐
f) I've been dealing with problems well	☐	☐	☐	☐	☐
g) I've been thinking clearly	☐	☐	☐	☐	☐
h) I've been feeling good about myself	☐	☐	☐	☐	☐
i) I've been feeling close to other people	☐	☐	☐	☐	☐
j) I've been feeling confident	☐	☐	☐	☐	☐
k) I've been able to make up my own mind about things	☐	☐	☐	☐	☐
l) I've been feeling loved	☐	☐	☐	☐	☐
m) I've been interested in new things	☐	☐	☐	☐	☐
n) I've been feeling cheerful	☐	☐	☐	☐	☐

56. Which of the following best describes your walking speed? *(Tick one box)*

a) Unable to walk ☐ b) Very slow ☐ c) Stroll at an easy pace ☐

d) Normal pace ☐ e) Fairly brisk ☐ f) Fast ☐

57. Have you had any falls in the past 12 months? *(Tick one box)*

a) No falls ☐ b) One fall ☐ c) More than one fall ☐

Section Three: Health *(continued)*

58. Do you have problems with your memory? *(Tick one box)*

a) No problems ☐ b) Sometimes, but not a serious problem ☐ c) serious problems ☐

59. Do you think your memory has got worse over the past 2 years? *(Tick one box)*

a) No ☐ b) A bit worse ☐ c) A lot worse ☐

60 Do you have difficulty with any of the following activities? *(One tick for each row)*

		No problem	Mild Problem	Moderate Problem	Severe Problem
a)	Walking	☐	☐	☐	☐
b)	Getting up from sitting	☐	☐	☐	☐
c)	Opening jars that have never been opened	☐	☐	☐	☐

61. Below is a list of <u>problems that people sometimes have</u>. Please read each one carefully and tick the box that best describes how much that problem has distressed or bothered you during the <u>past 7 days</u> including today *(One tick for each row)*

		Not at all	A little bit	Moderately	Quite a bit	Extremely
a)	Faintness or dizziness	☐	☐	☐	☐	☐
b)	Pains in the heart or chest	☐	☐	☐	☐	☐
c)	Nausea or upset stomach	☐	☐	☐	☐	☐
d)	Trouble getting your breath	☐	☐	☐	☐	☐
e)	Hot or cold spells	☐	☐	☐	☐	☐

62. How much have you been troubled by the following sleep problems <u>in the past 3 months</u>?
(One tick for each row)

		No problem	Mild Problem	Moderate Problem	Severe Problem
a)	Difficulty falling asleep	☐	☐	☐	☐
b)	Difficulty staying asleep	☐	☐	☐	☐
c)	Waking up too early	☐	☐	☐	☐
d)	Not feeling refreshed in the morning	☐	☐	☐	☐

63. Below is a list of ways you might have felt or behaved – please tell us how often you have felt this way during the past 7 days including today *(One tick for each row)*

		During the past 7 days			
		Rarely or none of the time (less than one day)	Some or a little of the time (1-2 days)	Occasionally or a moderate amount of the time (3-4 days)	Most or all of the time (5-7 days)
a)	I was bothered by things that usually didn't bother me	☐	☐	☐	☐
b)	I did not feel like eating; my appetite was poor	☐	☐	☐	☐
c)	I felt that I could not shake off feeling low, even with help from my family and/or friends	☐	☐	☐	☐
d)	I felt I was just as good as other people	☐	☐	☐	☐
e)	I had trouble keeping my mind on what I was doing	☐	☐	☐	☐
f)	I felt depressed	☐	☐	☐	☐
g)	I felt that everything I did was an effort	☐	☐	☐	☐
h)	I felt hopeful about the future	☐	☐	☐	☐
i)	I thought my life had been a failure	☐	☐	☐	☐
j)	I felt fearful	☐	☐	☐	☐
k)	My sleep was restless	☐	☐	☐	☐
l)	I was happy	☐	☐	☐	☐
m)	I talked less than usual	☐	☐	☐	☐
n)	I felt lonely	☐	☐	☐	☐
o)	People were unfriendly	☐	☐	☐	☐
p)	I enjoyed life	☐	☐	☐	☐
q)	I had crying spells	☐	☐	☐	☐
r)	I felt sad	☐	☐	☐	☐
s)	I felt that people dislike me	☐	☐	☐	☐
t)	I could not get "going"	☐	☐	☐	☐

Section Three: Health *(continued)*

Past 12 months

64. In the past 12 months have you lost more than 10 pounds (4.5 kg) *unintentionally (i.e. without dieting or exercise)?*

a) Yes ☐ b) No ☐

65. During the <u>past 12 months</u>, have you had pain in your BACK or NECK for a month or longer that made it difficult or impossible to get washed or dressed or do household chores?

a) No ☐ b) Yes ☐

66. During the <u>past 12 months</u>, have you had pain in your ARM(S) or SHOULDER(S) for a month or longer that made it difficult or impossible to get washed or dressed or to do household chores?

a) No ☐ b) Yes ☐

67. During the <u>past 12 months</u>, have you had pain in your LEG(S) for a month or longer that made it difficult or impossible to get washed or dressed or do household chores?

a) No ☐ b) Yes ☐

68. During the <u>past 12 months</u>, how many days have you had off work in total because of problems with your health? *(Tick one box)*

a) No time ☐ b) Less than 5 days ☐ c) 5 to 20 days ☐

d) More than 20 days ☐ or e) Not applicable *(not working over this time)* ☐

69. During the <u>past 12 months</u>, how many days have you had off work in total because of pain in your back, neck, arms, shoulders or legs? *(Tick one box)*

a) No time ☐ b) Less than 5 days ☐ c) 5 to 20 days ☐

d) More than 20 days ☐ or e) Not applicable *(not working over this time)* ☐

70. During the <u>past 12 months</u>, have you had to cut down, avoid or change what you normally do at work because of health problems? *(Tick one box)*

a) Yes, a lot ☐ b) Yes, a little ☐ c) No, not at all ☐

d) Not applicable *(not working over this time)* ☐

You have now finished. Please place this form in the pre-paid envelope supplied and post it back to us

THANK YOU!
